# Clinical Feasibility and Surgical Outcomes of a 3D-Printed Template-Based PMMA Implant Workflow for Genioplasty

**DOI:** 10.3390/jcm15093294

**Published:** 2026-04-26

**Authors:** Sunje Kim, Young Mook Yun, Chunghun Ha, Da Hyun Kang, Sabeom Park

**Affiliations:** 1 Department of Plastic and Reconstructive Surgery, Chungnam National University Hospital, Daejeon 35015, Republic of Korea; ksj9243@gmail.com; 2 Face Plastic Surgery, 203 Daedeok-daero, Seo-gu, Daejeon 35345, Republic of Korea; 3 Department of Industrial and Data Engineering, Hongik University, Seoul 04066, Republic of Korea; chunghun.ha@hongik.ac.kr; 4 Department of Internal Medicine, Chungnam National University Hospital, Chungnam National University College of Medicine, Daejeon 35015, Republic of Korea; ibelieveu113@cnuh.co.kr; 5 Center of Biohealth Convergence and Open Sharing System, Hongik University, 94 Wausan-ro, Mapo-gu, Seoul 04066, Republic of Korea

**Keywords:** genioplasty, 3D printing, polymethyl methacrylate

## Abstract

**Background:** Achieving facial harmony in patients with micrognathia requires precise chin augmentation. While conventional ready-made implants often fail to conform to unique mandibular surfaces, expensive patient-specific options like PEEK or Titanium lack intraoperative adjustability. We introduce an innovative, cost-effective workflow utilizing 3D-printed templates to fabricate customized Polymethyl Methacrylate (PMMA) implants, emphasizing their clinical feasibility and intraoperative versatility. **Methods:** We retrospectively analyzed 20 patients with mild-to-moderate micrognathia (<6 mm advancement) who underwent genioplasty between March 2021 and June 2022. Patient-specific templates were produced via Fused Deposition Modeling (FDM) using low-shrinkage Acrylonitrile Butadiene Styrene (ABS) filament. During surgery, final PMMA implants were molded using these sterilized templates. Accuracy was evaluated by comparing mental advancement across preoperative, virtual simulation, and 6-month postoperative stages using Vectra 3D scanning. **Results:** Quantitative analysis revealed high fidelity between virtual planning and clinical outcomes. The mean discrepancy in horizontal advancement was only 1.02 mm (Planned: 5.04 mm vs. Actual: 4.02 mm). Statistical analysis showed a strong positive correlation (r = 0.928, *p* = 0.001). Subjective patient satisfaction was high, with 90% reporting “exceptional” or “very improved” results on the Global Aesthetic Improvement Scale (GAIS). Two cases of transient numbness resolved spontaneously within two months. **Conclusions:** This workflow combines FDM-based template fabrication with intraoperative PMMA molding, enabling real-time adjustment of implant geometry. The results demonstrate a high level of agreement between virtual planning and postoperative outcomes, supporting the clinical reliability of this approach. It may serve as a practical alternative to conventional CAD/CAM methods, particularly in cases requiring both precision and intraoperative flexibility.

## 1. Introduction

While perspectives on facial aesthetics vary, the profile extending from the forehead and nose to the chin remains a critical determinant of facial harmony. From an aesthetic standpoint, micrognathia (a receding chin) often disrupts this balance, frequently serving as a significant source of psychological stress for patients. Various procedures, including osseous genioplasty, injectable fillers, and autologous fat grafting, have been employed to address this concern [1,2]. Fillers offer a simple and non-invasive solution but are limited by their temporary nature due to degradation. Similarly, while autologous fat grafting is relatively safe, it is often hindered by unpredictable resorption rates and the inability to replicate the hardness and texture of natural bone.

Consequently, osseous genioplasty and alloplastic implant insertion are considered the most definitive methods for chin augmentation. Osseous genioplasty, often combined with other orthognathic procedures, is typically reserved for severe micrognathia or cases involving significant malocclusion [3]. However, for most patients seeking purely cosmetic improvement, chin augmentation using implants remains the preferred choice [4].

Although autogenous bone grafting (e.g., using the iliac crest) is an alternative, it is less frequently performed due to donor-site morbidity, including additional pain and scarring [5].

The most widely utilized alloplastic materials for chin augmentation include Medpore [6], Silicone [7], and Gore-Tex [8]. Opinions regarding the clinical superiority and aesthetic outcomes of these materials vary among surgeons [9]. A significant limitation of these conventional materials is the difficulty in customization within a standard hospital environment; surgeons often rely on mass-produced, ready-made implants. Such implants are challenging to contour precisely to the unique bony surfaces of individual patients. Furthermore, vague preoperative explanations regarding postoperative changes can lead to a discrepancy between patient expectations and surgical outcomes, resulting in dissatisfaction.

To overcome these challenges, 3D printing has recently been integrated into genioplasty. Various additive manufacturing techniques have been applied in maxillofacial surgery, including stereolithography (SLA), selective laser sintering (SLS), and fused deposition modeling (FDM) [10]. SLA and SLS offer high resolution and enable the use of advanced materials; however, they are often associated with higher production costs and longer fabrication times. In contrast, FDM is more accessible and cost-effective, making it suitable for routine clinical applications despite its relatively lower resolution. In terms of materials, high-performance options such as titanium and polyether ether ketone (PEEK) have been widely used for patient-specific implants due to their mechanical strength and biocompatibility [11]. However, these materials are typically fabricated as rigid preoperative constructs, limiting intraoperative adaptability. Previous studies have reported that 3D-printed surgical guides and template-based workflows generally demonstrate discrepancies within the submillimeter to low-millimeter range, indicating acceptable but variable accuracy depending on the fabrication method [12,13].

While several studies have confirmed that 3D printing enhances surgical accuracy and efficiency—primarily through preoperative virtual simulation, intraoperative surgical guides, or customized plate design—its application in direct implant fabrication remains limited [14]. Despite these advancements, most existing workflows rely on either fully pre-fabricated implants or indirect template-based approaches with limited intraoperative adaptability. The novelty of this study lies in integrating template-based fabrication with intraoperative PMMA molding, enabling real-time adjustment of implant geometry while maintaining the advantages of digital preoperative planning.

This study aims to present a comprehensive approach involving preoperative and postoperative 3D analysis, the fabrication of patient-specific implant templates using 3D printing, and the intraoperative processing and application of PMMA implants. The null hypothesis (H_0_) of this study was that there would be no statistically significant difference in the degree of mental advancement between the preoperative virtual simulation and the actual 6-month postoperative outcome.

## 2. Materials and Methods

### 2.1. Research Procedure

This study was conducted to evaluate the accuracy of a workflow for fabricating patient-specific polymethyl methacrylate (PMMA) implants using 3D-printed templates in genioplasty. The research was designed as a case series involving a consecutive cohort of patients treated between March 2021 and June 2022.

A total of 20 patients with micrognathia were included, specifically targeting those who did not require or desire mental advancement exceeding 6 mm. Patients with concurrent severe systemic illnesses were excluded from the study. The study protocol received approval from the Institutional Review Board (approval no. 2022-06-107), and written informed consent was obtained from all subjects prior to their enrollment.

**Figure 1:**
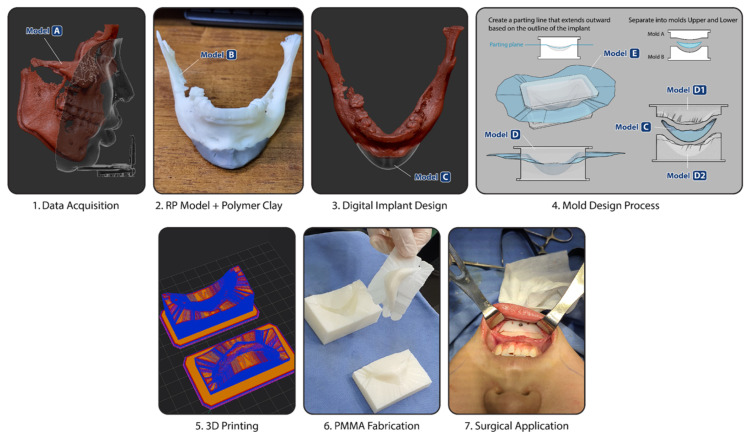
Workflow of the template-based PMMA implant fabrication for genioplasty. The process includes data acquisition and fusion, physical modeling using polymer clay, digital implant design, mold fabrication, 3D printing, PMMA implant fabrication, and surgical application.

The comprehensive workflow comprises the following sequential stages:

(1) Image acquisition and diagnosis, (2) Data digitization, (3) 3D modeling and virtual surgical planning, (4) 3D printing, (5) Implant fabrication, and (6) Surgical application and outcome assessment. This entire procedure is schematically illustrated in Figure 1.

### 2.2. Digitization Process

The patients’ three-dimensional (3D) facial soft tissue data were acquired using the Vectra 3D system (Canfield Scientific, Parsippany, NJ, USA). To analyze mandibular morphology, cone-beam computed tomography (CBCT) was performed using the Vatech Expert X18 system (PHT-76CHS, Vatech Co., Ltd., Hwaseong, Republic of Korea). The image data were stored in Digital Imaging and Communications in Medicine (DICOM) format.

CBCT scans were performed under high-resolution conditions with a field of view of 18 × 15 cm and a voxel size of 0.20 mm. The images were acquired using centered positioning and occlusion-based vertical alignment. Subsequently, all data were aligned within a unified coordinate system for 3D analysis.

### 2.3. Data Processing

The acquired DICOM data were reconstructed into a three-dimensional model using Mimics software 21.0 (Materialise, Leuven, Belgium). Mandibular segmentation was performed utilizing the thresholding function, with values set between 226 and 3071 Hounsfield units (HU) to selectively extract the bone based on its density. Subsequently, manual refinements were applied to ensure the anatomical accuracy of the reconstructed structures. The finalized mandibular model (Model-A) was converted into a stereolithography (STL) format and fabricated as a physical rapid prototyping (RP) model via Fused Deposition Modeling (FDM)-based 3D printing. Subsequently, polymer clay (Sculpey3, Gray, 57 g) was manually applied to the RP model to simulate the desired implant morphology in accordance with the planned mental advancement. The clay-added model was then digitized through CT scanning to generate a new 3D dataset, defined as Model-B. Model-A and Model-B were aligned in 3-matic (Materialise, Leuven, Belgium) using a two-step registration process, beginning with N-point registration for initial alignment, followed by global registration to minimize residual discrepancies.

This approach was chosen to prevent the ‘averaging errors’ frequently encountered in global best-fit methods when aligning asymmetrical or surgically altered structures. Following this primary alignment, Global Registration (Best-fit) was applied to minimize residual surface discrepancies and ensure overall geometric accuracy.

The registered models were further adjusted to ensure left-right symmetry based on the symphyseal midline and the Frankfort horizontal plane. Finally, both datasets were imported into ZBrush 4R7 (Pixologic, Los Angeles, CA, USA), where Model-B served as a reference to design the customized chin implant on Model-A. The final implant model (Model-C) was generated through a subtractive process, ensuring precise anatomical adaptation to the patient’s mandibular contour.

### 2.4. 3D Printing Process

The designed implant templates and molds were fabricated using a Fused Deposition Modeling (FDM)-based 3D printer (CUBICON Style Plus–A15D, CUBICON Co., Ltd., Seongnam-si, Republic of Korea) and its proprietary software, Cubicreator4 V4.1.0. This device provides a high printing precision of ±0.1 mm, making it suitable for precise dental and maxillofacial applications.

To ensure the structural integrity of the molds and mitigate potential thermal deformation during the intraoperative exothermic curing of PMMA, a low-shrinkage ABS-A100 filament was specifically selected. Printing was performed using the manufacturer’s dental presets (Style Plus-A15D, ABS-A100, 0.05 mm layer), which are optimized for high-fidelity anatomical models. The primary printing parameters included a layer thickness of 0.05 mm, while infill density, nozzle temperature, and bed temperature were set according to the manufacturer-recommended preset values.

Following the printing process, the templates underwent surface refinement to ensure smooth contours. Prior to clinical use, the templates were sterilized using Ethylene Oxide (EtO) gas, a low-temperature sterilization method chosen to prevent the thermal warping associated with high-temperature autoclaving.

### 2.5. Implant Tray Fabrication

Based on the finalized customized chin implant model (Model-C), a mold system was designed for implant fabrication. Using ZBrush, a solid block fully enclosing the implant geometry was created. This block was then divided along a reference plane into two separate components, resulting in a separable mold system consisting of an upper and a lower part. Subsequently, the negative cavities corresponding to the implant shape were generated through a subtractive process to create the final mold structure.

### 2.6. Surgical Application

For anesthesia, a mental nerve block was combined with local infiltration using a 1:100,000 epinephrine solution. The surgical approach involved a standard transverse intraoral labial incision. A subperiosteal pocket was dissected to a size sufficiently larger than the planned implant to ensure tension-free placement, with careful attention to avoid injury to the mental nerve.

Following meticulous hemostasis, a thin layer of a petroleum-based separating agent (Vaseline) was applied to the surface of the pre-fabricated template. This application served a dual purpose: it acted as a lubricant for easy separation and provided a chemical barrier to prevent the MMA monomer from acting as a solvent on the ABS surface, thereby ensuring material integrity. To avoid thermal deformation, the ABS templates were sterilized using a low-temperature method (Ethylene Oxide) rather than high-temperature autoclaving, considering the glass transition temperature of the material. The PMMA resin was prepared and poured into the template. To further mitigate the risk of thermal warping from the exothermic curing of PMMA, the implant was carefully molded and separated from the template during the ‘dough stage’ before reaching its peak exothermic temperature.

After approximately one minute, the PMMA hardened and was separated from the template. The resulting implant was then copiously rinsed with sterile saline solution to remove any residual monomers and surface debris. After confirming the integrity of the implant, excess material was trimmed as necessary.

The customized implant was then inserted into the pre-formed subperiosteal pocket. If required, additional refinement was performed using a surgical burr or rasp.

The implant was carefully aligned with the patient’s symphyseal midline and fixed securely using an 8–11 mm titanium screw. The mucosal incision was closed with sutures, and patients were typically discharged on the same day. Postoperative care included compressive taping for five days and a seven-day course of oral antibiotics.

## 3. Results

The study cohort consisted of 20 patients (16 females and 4 males) with a mean age of 25.2 years. None of the patients had a significant past medical history.

### 3.1. Assessment of Patient Satisfaction

At the 6-month follow-up assessment, the patients were asked to rate their satisfaction with the outcomes classified as Global Aesthetic Improvement Scale (GAIS) (Table 1).

### 3.2. Outcome Assessment and Statistical Analysis

**Figure 2:**
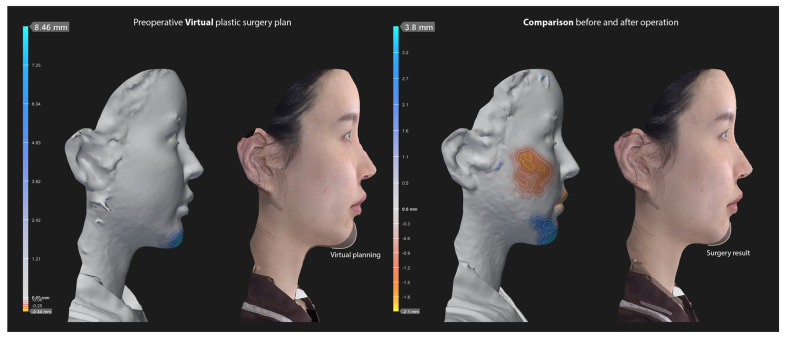
Virtual surgery plan using vertra (**left**) and comparison image of chin contour and advancement degree after actual surgery (**right**).

Advanced horizontal changes in virtual surgical planning and actual surgery results at postoperative 6 months were assessed using preoperative and postoperative virtual imaging. (Figure 2). Statistical analyses were performed using SPSS ver. 12.0 for Windows (SPSS Corp., Armonk, NY, USA). Statistical significance was set at *p* < 0.05.

**Figure 3:**
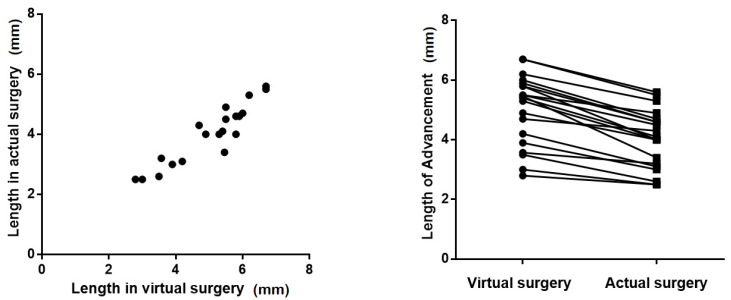
Comparison of advancement length between virtual surgical planning and actual surgical outcomes. (**left**) Scatter plot demonstrating the correlation between planned and achieved advancement (r = 0.928, *p* = 0.001). (**right**) Paired comparison of advancement length between virtual surgery and actual surgery for each patient.

In the Vectra-based virtual simulation, the average planned advancement of the pogonion was 5.04 mm (range: 2.8–6.7 mm). Following the actual surgical procedure, the postoperative assessment showed a mean advancement of 4.02 mm (range: 2.5–5.6 mm). The average discrepancy between the simulated and actual advancement was 1.02 mm (Table 2). Statistical analysis revealed a Pearson correlation coefficient of 0.928 (*p* = 0.001), indicating a strong positive correlation and high concordance between the virtual planning and clinical outcomes (Figure 3).

**Figure 4:**
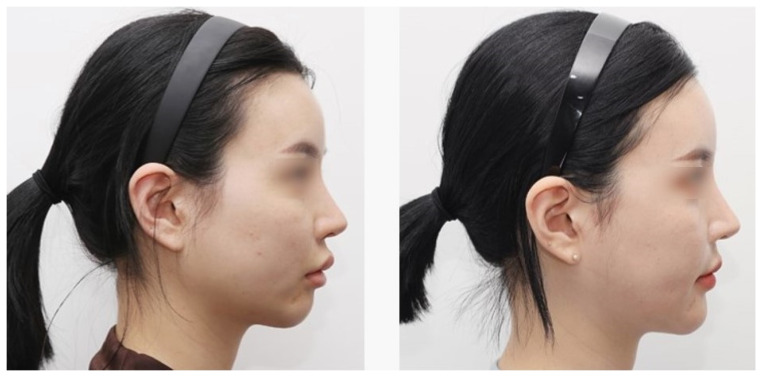
Case 1: Preoperative lateral view (**left**) and postoperative 6-month lateral view (**right**) of a 25-year-old woman who had micrognathia. PMMA implant using 3D printing template technique was performed. It shows a naturally corrected chin contour.

**Figure 5:**
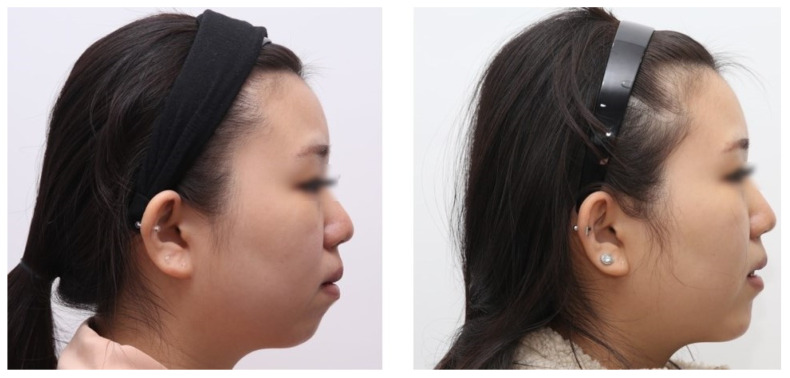
Case 2: Preoperative lateral view (**left**) and postoperative 6-month lateral view (**right**) of a 23-year-old woman who had micrognathia. The same 3D printing template method was used, and the degree of chin advancement was satisfactorily changed to match the overall facial profile.

Subjective patient satisfaction was high across all 20 cases, with 13 reporting “exceptional improvement,” 5 “very improved,” and 2 “improved.” These satisfactory results were consistent with clinical photographic evaluations and the operator’s aesthetic assessment (Figure 4 and Figure 5). Regarding postoperative complications, two patients experienced localized numbness in a small area of the lower lip; however, both cases resolved spontaneously within two months of follow-up. No other major complications, such as permanent paresthesia, infection, implant displacement, or protrusion, were observed.

## 4. Discussion

The inception of 3D printing is widely attributed to the pioneering work of Charles Hull and 3D Systems Corp [15]. Since then, the market has expanded rapidly, primarily within the automotive, aerospace, and maritime industries. In the biomedical field, 3D printing—specifically Fused Deposition Modeling (FDM)—has seen accelerated adoption since the early 2000s. This process, which involves melting and layering solid filaments, has been instrumental in manufacturing biocompatible thermoplastic scaffolds for tissue engineering [16].

Beyond tissue engineering, 3D printing is now extensively utilized in surgical simulations, custom implant fabrication, and the development of prosthetic devices such as hearing aids and dentures. A prominent application in craniofacial surgery includes rhinoplasty and cranioplasty. By considering an individual’s unique cartilaginous and bony anatomy, surgeons can pre-manufacture customized implants, thereby reducing operative time and enhancing both surgical efficiency and patient satisfaction [17,18]. Our study draws significant parallels with skull reconstruction techniques, where PMMA is frequently employed to repair defects resulting from trauma, tumors, or hemorrhage. Recent advancements have made it possible to fabricate both templates and patient-specific implants using 3D printing [19,20]. While 3D printing has also been applied to mandibular reconstruction, its use has often been limited by cost and production time, focusing primarily on fibular flap guides or preoperative plate contouring [21,22]. In genioplasty, the technology is emerging but remains largely confined to preoperative simulations and surgical guides rather than direct implant fabrication [14,23].

In the present study, we observed a high level of agreement between virtual surgical planning and actual postoperative outcomes, suggesting that the proposed workflow can achieve reliable and clinically meaningful results. In relation to the predefined null hypothesis, these findings indicate close agreement between planned and achieved outcomes, supporting the clinical validity of the workflow without implying formal statistical equivalence.

From a clinical perspective, the observed mean discrepancy of 1.02 mm can be considered relatively small and within an acceptable range for surgical accuracy. Previous studies have suggested that minor deviations of this magnitude are generally compatible with satisfactory clinical outcomes in genioplasty procedures [24,25]. This discrepancy may be attributed to both technical and biological factors. Mechanistically, intraoperative manual molding of PMMA within the 3D-printed template may introduce subtle volumetric variations during polymerization, including the effects of exothermic curing and material shrinkage. In addition, the accuracy assessment in this study was based on Vectra 3D scanning, which reflects soft tissue contour changes rather than static bone position. Therefore, the final postoperative outcome incorporates individual variability in soft tissue remodeling and thickness, which are inherent biological factors. Importantly, the high level of patient satisfaction observed in this study suggests that such discrepancies are unlikely to have a significant impact on aesthetic outcomes in clinical practice.

These findings may be particularly relevant given the complex anatomical characteristics of the facial skeleton. In this context, the cranial vault has relatively simple curvatures, making 3D design straightforward. In contrast, the midface and lower third of the facial skeleton possess complex, multi-axial contours that demand high-precision 3D modeling. Furthermore, even the most precisely manufactured implants must account for intraoperative variables, such as excessive scar tissue in secondary cases, individual skin elasticity, and overall profile changes when concurrent procedures like rhinoplasty or fat grafting are performed. These variables often necessitate intraoperative reshaping or manipulation of the implant. This concordance between planned and postoperative outcomes may be explained by the combination of precise digital preoperative planning and the intraoperative adaptability of PMMA. Unlike fully prefabricated implants, intraoperative molding allows fine adjustment to patient-specific anatomical and soft tissue conditions, which may help minimize cumulative discrepancies across the workflow. This approach may also reduce error propagation across multiple workflow stages, as intraoperative adjustments allow correction of minor discrepancies introduced during imaging, segmentation, or template fabrication.

Previous studies on 3D printing-assisted genioplasty have primarily focused on surgical guides or preoperative simulations, reporting clinically acceptable accuracy within a comparable range [12,13,14]. Our findings are generally consistent with these reports, while extending previous approaches by incorporating intraoperative implant fabrication, which may enhance surgical flexibility without compromising overall accuracy.

While various materials are available for craniofacial reconstruction (Table 3), the anatomical characteristics of the chin require a material that maintains structural integrity while remaining workable.

While advanced manufacturing techniques such as Stereolithography (SLA) and Selective Laser Sintering (SLS) offer superior resolution and the ability to process high-performance materials like Titanium or Polyether ether ketone (PEEK), their high cost and the inherent rigidity of the final products often limit their practical utility in routine aesthetic procedures [10]. In contrast to these rigid implants, which preclude any significant intraoperative modification once fabricated, the use of FDM-printed ABS templates for PMMA casting allows for real-time adjustments using standard surgical tools [11]. This hybrid workflow provides a pragmatic balance between digital precision and the clinical flexibility required to account for unpredictable patient-specific variables, such as soft tissue tension or minor anatomical discrepancies discovered during surgery (Table 3) [26,27]. Importantly, the ability to perform intraoperative adjustments may provide a critical advantage in managing patient-specific anatomical variability, which is difficult to address using fully prefabricated implants. Based on our findings, PMMA combined with 3D-printed templates may represent a clinically practical and adaptable option for customized genioplasty.

Complications in chin augmentation can include asymmetry due to improper placement, hematoma, infection, and, rarely, mental nerve compression or bone resorption [28]. While hematoma and infection risk can be minimized through meticulous hemostasis and perioperative care, bone resorption is often linked to increased pressure from oversized implants [29]. Such implants also heighten the risk of displacement or extrusion [30]. 3D printing addresses these issues by allowing surgeons to select an optimally sized implant through virtual surgery and patient consultation. Because these implants are engineered to fit the patient’s unique bony base, they exhibit minimal postoperative movement, promote early stability, and prevent step-deformities, thereby reducing foreign body sensations.

Despite its advantages, this study has several limitations. First, patients requiring mental advancement exceeding 6 mm were excluded, as osseous genioplasty is generally considered more appropriate for severe micrognathia. Therefore, our findings are limited to mild-to-moderate cases. Second, this study was conducted as a retrospective case series with a relatively small sample size, which may limit the generalizability of the results. In addition, the accuracy assessment was based on the comparison between virtual planning and postoperative outcomes, without stage-by-stage error analysis, and thus the contribution of each intermediate step could not be fully evaluated. Finally, although the workflow is clinically feasible, it involves multiple steps that may be technique-sensitive and require collaboration with experienced personnel. The mold-based process may also introduce minor deviations; however, future developments in direct PMMA 3D printing may further simplify the workflow and reduce potential sources of error.

## 5. Conclusions

In conclusion, the integration of virtual surgical planning, 3D-printed templates, and intraoperative PMMA fabrication offers a precise and clinically accessible approach to genioplasty. Our findings demonstrate high concordance between simulated plans and actual surgical results, confirming that this digital-to-analog workflow can achieve predictable mental advancement.

The primary innovation of this method lies in its intraoperative adaptability. Unlike high-performance rigid materials such as PEEK or Titanium, which preclude real-time modification, the excellent machinability of PMMA allows for bedside refinements using a burr or rasp. This flexibility ensures superior anatomical conformity to the patient’s unique mandibular surface, effectively accounting for unpredictable intraoperative variables such as soft-tissue tension and subperiosteal pocket dimensions.

The high rate of patient satisfaction combined with the absence of major complications validates the safety and aesthetic efficacy of this approach for mild-to-moderate micrognathia. As a cost-effective and high-fidelity alternative to conventional ready-made implants, this technique suggests promising potential for broader applications in various facial bone reconstructions and aesthetic maxillofacial procedures.

## Figures and Tables

**Table 1:** Global Aesthetic Improvement Scale scores.

**Degree**	**Description**
1 Exceptional improvement	Excellent corrective result
2 Very improved patient	Marked improvement of the appearance, but not complete
3 Improved patient	Improvement of the appearance, better compared with the initial condition, but a touch-up is advised
4 Unaltered patient	The appearance substantially remains the same compared with the original condition
5 Worsened patient	The appearance has worsened compared with the original condition

**Table 2:** Patients’ Summary (Advancement of pogonion position in virtual planning and actual surgery).

Patients	Advancement of Pogonion in Virtual Surgery (A)	Advancement of Pogonion in Actual Surgery (B)	Difference (A–B)
1	5.5 mm	4.9 mm	0.6 mm
2	3.0 mm	2.5 mm	0.5 mm
3	4.7 mm	4.3 mm	0.4 mm
4	3.5 mm	2.6 mm	0.9 mm
5	4.9 mm	4.0 mm	0.9 mm
6	5.5 mm	4.5 mm	1.0 mm
7	5.8 mm	4.0 mm	1.8 mm
8	6.7 mm	5.5 mm	1.2 mm
9	4.2 mm	3.1 mm	0.9 mm
10	5.3 mm	4.0 mm	1.3 mm
11	6.2 mm	5.3 mm	0.9 mm
12	6.0 mm	4.7 mm	1.3 mm
13	3.9 mm	3.0 mm	0.9 mm
14	5.4 mm	4.1 mm	1.3 mm
15	5.5 mm	3.4 mm	2.1 mm
16	6.7 mm	5.6 mm	1.1 mm
17	3.5 mm	3.2 mm	0.3 mm
18	2.8 mm	2.5 mm	0.3 mm
19	5.9 mm	4.6 mm	1.3 mm
20	5.8 mm	4.6 mm	1.2 mm
Average	5.04 mm	4.02 mm	1.02 mm

**Table 3:** Comparison of Physical and Clinical Properties of Common Dental Implant & Prosthetic Materials.

Biomaterial	Density (g/cm^3^)	Melting Point (°C)	Hardness	Machinability	Resorbability	Tensile Strength (Mpa)
PMMA	1.18	105	Moderate	Excellent	Non-resorbable	50–79
Titanium Alloy	4.43	1660	High	Poor	Non-resorbable	860–940
Stainless Steel	7.95	1400	High	Moderate	Non-resorbable	500–700
PEEK	1.32	343	High	Good	Non-resorbable	90–100
PLA	1.25	175	Moderate	Good	Resorbable	40–60
PCL	1.15	60	Low	Good	Resorbable	15–25

PMMA = Poly methyl methacrylate; PEEK = Polyether ether ketone; PLA = Polylactic acid; PCL = Polycaprolactone.

## Data Availability

The data presented in this study are available upon request from the corresponding author. The data are not publicly available due to patients’ privacy.
